# 15 Poverty and Frailty in Patients with Burn Injuries: Important but Unrelated

**DOI:** 10.1093/jbcr/irac012.019

**Published:** 2022-03-23

**Authors:** David L Wallace, Keturah Sloan, Deborah Williams, Jason Heard, David Greenhalgh, Tina L Palmieri, Soman Sen, Kathleen S Skipton Romanowski

**Affiliations:** University of California, Davis, Sacramento, California; UC Davis Firefighters Burn Institute Regional Burn Center, Sacramento, California; Shriners Hospitals for Children, Woodland, California; University of California - Davis, Sacramento, California; ; UC Davis and Shriners Hospitals for Children Northern California, Sacramento, California; UC Davis, Sacramento, California; UC Davis, Sacramento, California

## Abstract

**Introduction:**

Previous work has demonstrated that frailty predicts mortality and patient disposition in burn patients >50 years old. It is unknown to what extent poverty contributes to these outcomes. There has been no work demonstrating the interplay of these two variables on patients with burn injuries. The purpose of this study was to determine the relationship of frailty and poverty in burn patients over the age of 50, and their association with patient outcomes.

**Methods:**

A 9-year retrospective chart review from 2009-2018 of patients >50 years old admitted to an ABA verified burn center with acute burn injuries was completed. Patient demographics, burn characteristics, frailty scores and poverty levels were collected. Frailty scores were assigned using the Canadian Study of Health and Aging Clinical Frailty Scale (scored 1-7). Frailty was dichotomized with scores >5 being frail. Poverty data were obtained using zip code and US census data. Poverty level was categorized according to whether a patient came from a zip code that had >20% of people living in poverty. Descriptive statistics, univariate analysis, and multivariate analysis were completed to examine the relationship between frailty and poverty, as well as each variable independently on mortality and length of stay (LOS).

**Results:**

A total of 953 patients were included. Mean age was 63.5 + 10.4 years and 675 (70.8%) were male. Mean %TBSA was 11.4%+14.2% and mean frailty score was 3.8 + 1.2. Upon admission, mean poverty score was 17.3+ 8.7. The overall mortality rate was 8.8%. Univariate analysis demonstrated that non-survivors had significantly higher chances of living in poverty (p=0.02). Similarly, univariate analysis showed that non-survivors were more likely to have frailty scores of 5 or greater compared to survivors. Multivariate logistic regression confirmed relationship between poverty and mortality (<20% vs >20%, OR 0.47 95% CI 0.25-0.89) and frailty and mortality ( >5 vs 1-4, OR 2.9 95%CI 1.4-5.8). It also demonstrated that the combined variable of frailty and poverty was not significantly associated with mortality (Wald χ2 2.0, p=0.15). Neither poverty (< 20% vs >20%, p=0.26) nor frailty (1-4 vs >5, p=0.52) were associated with LOS. Both poverty and frailty were associated with a patient’s disposition destination (p=0.03; p< 0.0001). Univariate analysis did not show a significant correlation between poverty and frailty (p=0.08), though there was a trend towards significance.

**Conclusions:**

Poverty and frailty each independently predict mortality and discharge destination in burn patients >50, but they are not associated with LOS, and do not show a significant association with each other, nor a combined effect on mortality.

